# Human Fat-Derived Mesenchymal Stem Cells Xenogenically Implanted in a Rat Model Show Enhanced New Bone Formation in Maxillary Alveolar Tooth Defects

**DOI:** 10.1155/2020/8142938

**Published:** 2020-01-13

**Authors:** Andrew Wofford, Austin Bow, Steven Newby, Seth Brooks, Rachel Rodriguez, Tom Masi, Stacy Stephenson, Jack Gotcher, David E. Anderson, Josh Campbell, Madhu Dhar

**Affiliations:** ^1^Department of Biochemistry and Cellular and Molecular Biology, College of Arts and Sciences, University of Tennessee, Knoxville, TN 37916, USA; ^2^Department of Large Animal Clinical Sciences, College of Veterinary Medicine, University of Tennessee, Knoxville, TN 37996, USA; ^3^Department of Oral and Maxillofacial Surgery, University of Tennessee Medical Center, Knoxville, TN 37920, USA; ^4^Graduate School of Medicine, Department of Surgery, University of Tennessee, Knoxville, TN 37920, USA

## Abstract

**Background:**

Due to restorative concerns, bone regenerative therapies have garnered much attention in the field of human oral/maxillofacial surgery. Current treatments using autologous and allogenic bone grafts suffer from inherent challenges, hence the ideal bone replacement therapy is yet to be found. Establishing a model by which MSCs can be placed in a clinically acceptable bone defect to promote bone healing will prove valuable to oral/maxillofacial surgeons.

**Methods:**

Human adipose tissue-derived MSCs were seeded onto Gelfoam® and their viability, proliferation, and osteogenic differentiation was evaluated *in vitro*. Subsequently, the construct was implanted in a rat maxillary alveolar bone defect to assess *in vivo* bone healing and regeneration.

**Results:**

Human MSCs were adhered, proliferated, and uniformly distributed, and underwent osteogenic differentiation on Gelfoam®, comparable with the tissue culture surface. Data confirmed that Gelfoam® could be used as a scaffold for cell attachment and a delivery vehicle to implant MSCs *in vivo*. Histomorphometric analyses of bones harvested from rats treated with hMSCs showed statistically significant increase in collagen/early bone formation, with cells positive for osteogenic and angiogenic markers in the defect site. This pattern was visible as early as 4 weeks post treatment.

**Conclusions:**

Xenogenically implanted human MSCs have the potential to heal an alveolar tooth defect in rats. Gelfoam®, a commonly used clinical biomaterial, can serve as a scaffold to deliver and maintain MSCs to the defect site. Translating this strategy to preclinical animal models provides hope for bone tissue engineering.

## 1. Background

Several clinical studies show a need for stronger, faster, and more reliable bone formation in defects or fractures following surgery, disease, or trauma. Cell-based therapies offer the potential to overcome these challenges, especially in dental and craniofacial healing [1, 2]. This is specifically a challenge in cases of larger defects or defects that are of complex anatomical shapes and sizes and require strong, mature bone regeneration for future implants. Additionally, in the field of oral and maxillofacial medicine, a relatively simple tooth extraction procedure, if not controlled, can lead to significant complications, including infection and osteonecrosis. Residual ridge resorption, resulting in reduced buccolingual and apicocoronal aspects at the site of extraction, is another common phenomenon that causes physical and economic concerns in human patients [3]. Furthermore, tooth extraction procedures are considered to be a major risk factor for bisphosphonate-induced osteonecrosis of the jaw (ONJ) [4]. If not treated promptly, this disorder can lead to complex morbidities and the loss of the entire jaw bone. Hence, there is a need for therapies capable of regenerating healthy new bone after such procedures, and thus preventing further complications.

Bone tissue engineering strategies include the use of viable cells in conjunction with biomaterials or scaffolds. Several bone tissue engineering studies have shown preference for using naïve, adult mesenchymal stem cells (MSCs) instead of differentiated osteoblasts for bone formation applications. Cells alone or cells combined with biomaterials may offer advantages compared to the results associated with the use of allografts or autografts. Human MSCs (hMSCs) are naïve multipotent cells which can be isolated from any adult tissue, including the bone marrow, fat, cord blood, and dental pulp. Adult MSCs are capable of differentiation to adipocytes, myocytes, chondrocytes, and osteoblasts, with these stem cell properties having been demonstrated *in vitro* and *in vivo* [5–8]. MSCs are typically expanded in culture, evaluated for their characteristics, and induced to undergo osteogenic differentiation, *in vitro*. Subsequent to the expansion and characterization, they are transplanted *in vivo* for therapy. Their efficacy is influenced by the complex *in vivo* microenvironment as well as the cellular and molecular properties of MSCs. Human MSCs have been shown to demonstrate significant beneficial effects on bone healing and repair of the appendicular, axial, and craniomaxillofacial bones [9, 10].

Another important component of bone tissue engineering is the use of scaffolds or biomaterials capable of serving as a delivery vehicle and a containment agent to hold cells at the defect site *in vivo*. Several commercially available materials have been reported to deliver MSCs, including porous and gelatin-based scaffolds [11–13]. Even though there are a number of commercially available cell delivery materials, prohibitive factors, including high costs or technical challenges in application, restrict general use. Most importantly, an ideal bone regeneration scaffold, which is osteoinductive, osteoconductive, and osseointegrative has yet to be developed [14, 15]. As a result, autogenous bone grafts remain the gold standard.

Gelfoam®, a gelatin-based material, is commonly used as a contact hemostat in healthcare facilities. A porous, pliable, and cost-effective material, Gelfoam® is also referred to as hydrolyzed collagen and is comprised of a proteinous material, which is generally prepared by boiling skin, tendons, ligaments, and/or bones with water. Hence, Gelfoam® does not by itself demonstrates any osteogenic properties, and thus can be used to deliver and evaluate the effect of MSCs on bone healing without any confounding factors. A study showed promising results for Gelfoam® as a hMSC delivery vehicle by analyzing loading kinetics, cellular distribution, cellular density using several biochemical assays, and its biocompatibility using a rabbit joint model [16].

We hypothesized that hMSCs will readily attach and proliferate on degradable clinical grade Gelfoam® structures and that delivery of xenogeneic cells via this nonbioactive vehicle in a rat maxillary tooth extraction model will promote repair and restoration of bone tissue at defect sites. As the implanted material does not have inherent osteobiologic properties, bone tissue regeneration capacity of the examined treatment will allow for evaluation of the osteogenic potential of MSCs alone *in vivo*. Based on the potential of MSCs to differentiate toward multiple lineages, including bone, it is anticipated that the application of a reservoir of these naïve cells, maintained at the site of injury via a bioinert structure, will result in enhanced repair of damaged tissue.

## 2. Methods

### 2.1. Biochemicals and Disposables

All biochemicals, cell culture supplements, and disposable tissue culture supplies were purchased from Thermo Fisher Scientific unless otherwise noted.

### 2.2. Gelfoam® as a Scaffold Material

Commercially obtained Gelfoam®, Pfizer, is a purified gelatin material derived from porcine skin that is stored at 15-30°C until use (Pfizer USP, Michigan, USA). Materials for *in vitro* and *in vivo* experiments were cut to size from bulk sheets.

### 2.3. Isolation, *Ex Vivo* Expansion, and *In Vitro* Osteogenesis of Human Mesenchymal Stem Cells

Stromal vascular fraction of cells was obtained from human adipose tissue from patients undergoing panniculectomies in accordance to a protocol approved by the IRB at the University of Tennessee Medical Center. Informed client consent was obtained prior to the harvest. The hMSCs were isolated, *ex vivo* expanded, and induced to undergo osteogenesis as described earlier [17]. Briefly, the hMSCs were grown to 80–90% confluency and then harvested with 0.05% trypsin/EDTA for cryopreservation (80% FBS, 10% DMEM/F12, 10% DMSO), or split and seeded into new flasks for *in vitro* assays and expansion, respectively. All experiments were performed using cells from passage 2–6 in complete growth media (DMEM/F12, 1% penicillin-streptomycin/amphotericin B, 10% FBS).

MSCs obtained were confirmed for their identity by their morphology, potential to undergo trilineage differentiation, and expression of specific protein markers, using methods reported earlier [17].

*In vitro* experiments were performed on identical passage numbers of hMSCs seeded simultaneously on Gelfoam® and the tissue culture substrates. Growth and osteogenic differentiation of hMSCs on the two substrates were carried out simultaneously.

### 2.4. RNA Extraction, cDNA Synthesis, and qPCR

RNA was extracted from both control hMSC cultures, grown on a polystyrene coated tissue culture surface and Gelfoam®-embedded hMSCs at days 7 and 21 of differentiation. Total RNA was isolated using TRIzol extraction agent (Thermo Fisher) as per the manufacturer's protocol and as reported earlier [18]. Briefly, total RNA was prepared and further purified using a RNeasy mini kit (Qiagen); cDNA was prepared using a high-capacity cDNA reverse transcription kit (Applied Biosystems); and qPCR analysis of the expression of the bone-specific markers osteopontin (OPN) and osteocalcin (OCN) was carried out using SYBR green master mix (Thermo Fisher) with GAPDH serving as the housekeeping gene using MX3005P real-time PCR cycler (Agilent).

Several preliminary experiments were run to determine ideal qPCR protocol, PCR mix, and annealing temperatures. qPCR was run using ABsolute Blue qPCR Mix (Thermo Fisher Scientific), with each reaction comprising of 5.0 *μ*L cDNA solution, 12.5 *μ*L ABsolute Blue SYBR Green ROX, 5.0 *μ*L RNase Free Water, and 2.5 *μ*L of the appropriate primers. All primer sequences and PCR conditions were derived from a previously published report [5].

### 2.5. Animals and Surgical Procedure

8-10 week old mixed gender Sprague Dawley rats (*n* = 36) were commercially obtained (Harlan Laboratories).

Animal procedures were performed in accordance with a protocol approved by the University of Tennessee, Institutional Animal Care and Use Committee (IACUC). Bone defects were generated using procedures modified from those described earlier [19–21]. Briefly, rats under anesthesia were placed in a supine position, and the mandible was opened to expose the maxillary surface. 1^st^ and the 2^nd^ maxillary molars were removed from one side, and the resulting void spaces in the alveolar processes were then levelled using a microdrill to form a slot-shaped trough in which the scaffold could be readily implanted. Defects were washed thoroughly with sterile saline to remove residual tissue debris. Scaffold material with and without cells was firmly placed in each defect prior to closure of the site with resorbable sutures. The side opposite to the defect was left intact to serve as a reference during histological analysis. The rats were fed a soft gel (Nutra-Gel, Bio-Serv) throughout the study period to prevent damage to surgical sites by standard dry pellet form food. Animals were sacrificed at weeks 1, 4, and 12 after surgery. Rats were divided into two groups with 6 rats per group per time point. One group received Gelfoam® alone, while the other group was treated with Gelfoam® loaded with 1 × 10^6^ hMSCs, which were seeded onto Gelfoam® 30-60 minutes prior to implantation.

### 2.6. Histomorphometry

Samples were harvested after sacrifice and subjected to histomorphometric processing and analyses as reported earlier [18]. All bones were fixed in Decal A for at least 24 hrs, following which, they were immersed in Decal B for at least 48 hrs for decalcification. Subsequently, 5 *μ*m sagittal sections were obtained and stained with hematoxylin and eosin (H&E) and Masson trichrome for analysis.

H&E staining was used to subjectively evaluate adverse reaction, if any due to either the Gelfoam® or the Gelfoam®+hMSCs construct. Masson's trichrome staining was evaluated and quantitated using Fiji software [22]. Two micrograph images of each slide were taken at 2.5x. Images included both the region where the alveolar bone defect was created and the region of the corresponding contralateral intact tooth and alveolar bone. Image colors were split into channels and threshold was adjusted to generate binary masks highlighting bone tissue surface. Regions of interest (ROI) were identified by using the rectangular selection tool to set the parameters of the alveolar bone tissue where the intact tooth is shown rooted. This selection was then transferred to an analogous site on contralateral defect side to maintain equal area and shape of the measured region. The percentage of bone tissue area coverage (BTAC) for ROIs of each image was calculated (Equation (1)). For each rat, the percentage of bone tissue area coverage in the defect region was divided by that in the intact region to obtain a bone regeneration ratio (BRR) for each defect (Equation (2)). The bone regeneration of the control (Gelfoam® only) and the Gelfoam®+hMSC-treated rats was compared at each time point of sacrifice (1, 4, and 12 weeks).

(1) $$\begin{array}{c} \frac{P\times \text{Coun}{\text{t}}_{\text{B}}}{P\times \text{Coun}{\text{t}}_{\text{T}}}=\text{BTAC} \end{array}$$

Equation (1) is for determining bone tissue area coverage (BTAC) for a given binary image in which the tissue of interest has been set to the maximum value. The measured pixel count for maximum value pixels is divided by the total count of image pixels. This ratio represents the fractional area coverage of the tissue of interest.

(2) $$\begin{array}{c} \frac{{\text{BTAC}}_{\text{D}}}{{\text{BTAC}}_{\text{I}}}=\text{BRR} \end{array}$$

Equation (2) is for determining the bone regeneration ratio (BRR) of a given complimentary set of intact and defect images. The bone tissue area coverage (BTAC) for the defect image of the set (BTAC_D_) is divided by that of the intact image (BTAC_I_). The ratio represents the level of bone formation within the defect site as compared with that of the native structure.

### 2.7. Immunohistochemistry

Unstained histological sections were subjected to immunohistochemical (IHC) staining to detect and analyze expression of proteins associated with bone, collagen, and vasculature structure formation. OPN and fibronectin (FN) expression correlate to early bone formation and cellular attachment, respectively, while the hematopoietic stem cell marker, CD34, represents angiogenic functions. Paraffin-embedded sections for IHC staining were prepared according to a standard protocol (Abcam IHC Protocol). Briefly, samples were deparaffinized in xylenes and rehydrated using decreasing concentrations of ethanol, ending with washing in distilled water. Antigen retrieval was performed utilizing a heated target retrieval agent (DAKO). Samples were exposed to 1% Triton in PBS and subsequent protein blocking solution prior to addition of primary antibodies. Biotinylated secondary antibody solutions targeting primary antibody host species IgG were followed by the addition of streptavidin-horseradish peroxidase (HRP). A Nova Red (Vector) kit was then utilized to stain HRP-labeled surface proteins for analysis.

Imaging of IHC-stained slides was performed with a Leica DMi1 light microscope at 5x magnification. Captured images were combined utilizing a FIJI stitching plugin, designed by Dr. Preibisch, to generate full tissue section images.

### 2.8. Statistical Analyses

For the RT-qPCR gene expression analysis, expression levels of each gene were normalized with GAPDH, serving as a housekeeping gene. Gene expression of the tissue culture seeded hMSCs was evaluated to ensure the accuracy of the real-time PCR conditions. Gene expression fold levels of both the Gelfoam®+hMSCs and tissue culture seeded hMSCs groups' were analyzed at day 21 relative to day 7. Fold changes for each gene were calculated using the 2^-*ΔΔ*C^_T_ formula (Applied Biosystems). Data was statistically analyzed using Student's *t* test with *p* < 0.05.

For the quantitative analysis of the rat alveolar bone healing, the level of bone formation obtained from the BRR values was analyzed using two-way ANOVA to evaluate the time (weeks of treatment) and group (scaffold alone and scaffold with MSCs) effects. Post hoc multiple comparisons were performed with Tukey's adjustment. Statistical significance was set at *p* < 0.05. All analyses were conducted using SAS 9.4 TS1M4 for Windows 64x (SAS Institute Inc., Cary, NC).

## 3. Results

### 3.1. Progenitor Cells Isolated from the Stromal Vascular Fraction Are MSCs

**Figure 1:**
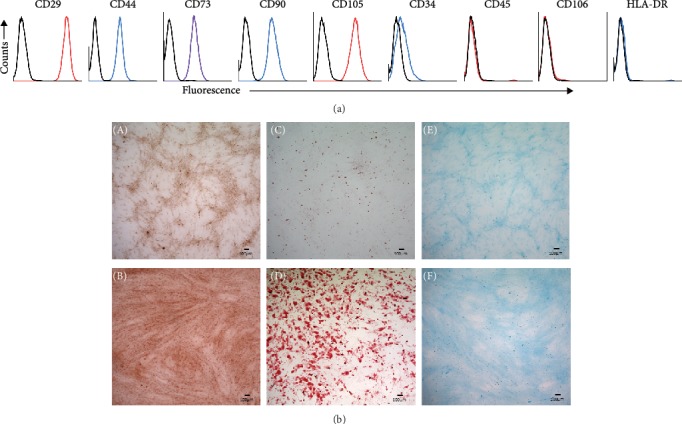
*In vitro* characterization of human MSCs. Immunophenotyping of hMSCs by flow cytometry (a) and trilineage differentiation (b). For immunophenotyping, hMSCs were stained with the indicated antibodies and then analyzed by flow cytometry. Cells strongly express the markers (CD29, CD44, CD73, CD90, CD105) associated with the MSCs, while expression of hematopoietic (CD34, CD45, HLA-DR) and endothelial (CD106) markers is markedly reduced. Black open histograms indicate isotype-matched controls for each antibody; colored open histograms represent positive reactivity. Trilineage differentiation assays of hMSCs shows representative images of alizarin red, oil-red-o, and Alcian blue staining of osteocytes (B), adipocytes (D), and chondrocytes (F), after *in vitro* differentiation. Corresponding undifferentiated hMSCs (A, C, E) are shown as controls.

Mesenchymal stromal cells were isolated from the stromal vascular fraction and subsequently expanded *ex vivo* to generate numbers sufficient for *in vitro* and *in vivo* applications. Prior to *in vivo* applications, the expanded cells were characterized *in vitro* to prove that they are indeed MSCs. We generated primary cultures of adipose tissue-derived hMSCs, which were characterized *in vitro* using methods described earlier [17]. Subjective evaluation demonstrated that the cells adhered to the tissue culture polystyrene surface and exhibited a fibroblast-like morphology during *in vitro* culturing and serial passaging. Using flow cytometry, cells were found to be >99.8% positive for CD29, CD44, CD73, CD90, and CD105. CD34 (hematopoietic), CD106 (endothelial), CD45 (leukocyte), and HLA-DR (MHC Class II) were detected at 28.3%, 4.26%, 2.43%, and 2.49%, respectively (Figure 1(a)). During passaging, the expression of CD34 significantly reduced to <5%, suggesting that serial passaging of hMSCs under the given cell culture conditions yielded a relatively homogenous culture of cells. Thus, the overwhelming majority of the cultured cells express the expected CD markers found on MSCs with minimal contamination of other cell types.

Additionally, we demonstrated the potential of hMSCs to differentiate into osteocytes, adipocytes, and chondrocytes (trilineage differentiation) *in vitro*. When isolated MSCs were induced with lineage-specific cocktail, they did undergo differentiation into the expected cell types compared to the controls, which were incubated in the absence of lineage-specific media. Alizarin red, oil-red-o, and Alcian blue staining confirmed the presence of calcium (osteocytes), lipids (adipocytes), and glycosaminoglycans (cartilage), respectively (Figure 1(b)). Therefore, the isolated progenitor cells met the specific criteria, and hence were indeed MSCs.

### 3.2. Cytocompatibility of Gelfoam®

**Figure 2:**
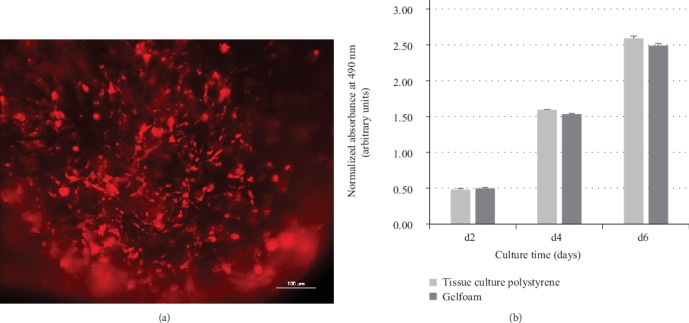
*In vitro* adherence and viability of hMSCs. The adherence and viability of hMSCs on Gelfoam® was evaluated using DiI imaging (a) and MTS assay (b). Representative image shows the red cytoplasmic fluorescence of hMSCs adhered to Gelfoam® after 6 days. A portion of the image is out of focus because of the 3D nature of the scaffold. Proliferation of hMSCs on Gelfoam® is comparable to cells seeded on tissue culture polystyrene surface for 2, 4, and 6 days. The absorbance at 490 nm is directly proportional to the number of living and proliferating cells. Tissue culture surface and Gelfoam® without any cells in the same culture conditions were used as blanks to obtain normalized values at each time point.

After proving the MSC nature, cells were seeded on Gelfoam® to verify cell adherence, distribution, and viability using DiI imaging (Figure 2(a)) and MTS proliferation assay for 6 days (Figure 2(b)), respectively. Detection of cell fluorescence and an observed linear increase in the absorbance with time indicated that cells were adhered to, were distributed uniformly on the material constructs, and that the viability and proliferation characteristics on Gelfoam® were similar to cultures grown on polystyrene surfaces. These results demonstrated that Gelfoam® is noncytotoxic, permits cell adherence, distribution, and does not hinder proliferation of MSCs. Furthermore, though Gelfoam® samples became pliable and spongy after media absorption, the matrix of the material did not lose its structural integrity within the study time period. As a result, Gelfoam® served as an effective means for containing cells after initial attachment.

### 3.3. hMSCs Maintain Osteogenic Capacity on Gelfoam®

**Figure 3:**
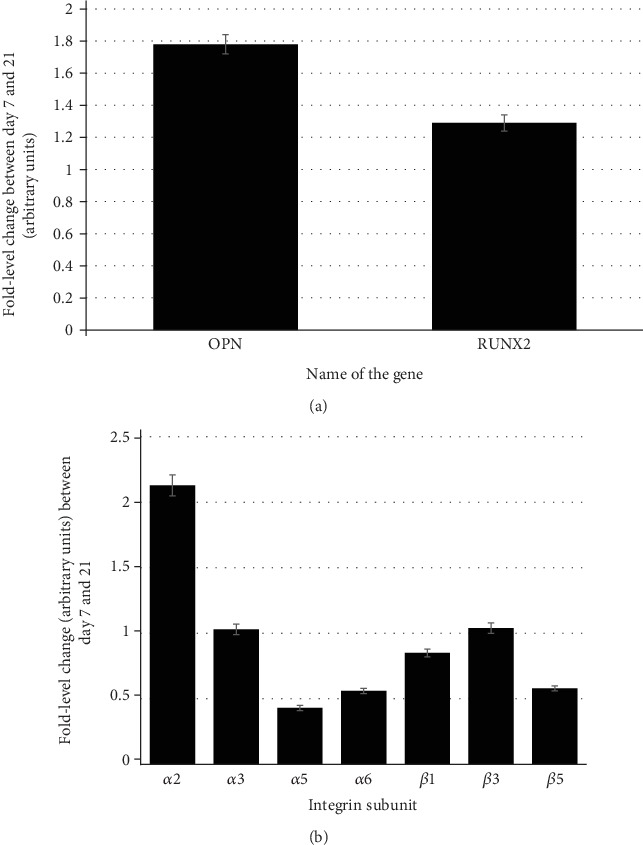
qPCR expression. Gene expression of the osteogenic-specific (a) and integrin subunit (b) genes. Relative fold differences in the expression of genes between days 7 and 21 during osteogenic differentiation of hMSCs on Gelfoam® were calculated using the delta-delta Ct method (Applied Biosystems). GAPDH was used as the housekeeping gene.

We next evaluated the osteogenic differentiation potential of hMSCs by profiling the expression of genes strongly associated with osteogenesis. We wanted to ensure that MSCs retained their osteogenic potential in presence of Gelfoam®. We used qPCR to assess the expression of two commonly used osteoblast markers, osteopontin (OPN) and the transcription factor, RUNX2 [23]. Quantitative PCR results of control cultures, i.e., hMSCs induced to undergo osteogenic differentiation on a tissue culture substrate, showed that both genes were expressed at both days 7 and 21 with significant upregulation of 4- and 2-fold difference, respectively, with time. Results confirmed that hMSCs underwent osteogenesis within this time period on the tissue culture polystyrene surface and that RNA extraction, cDNA synthesis, and PCR procedures were accurate. Using the parameters validated for the control cells, the expression of both markers was detected in Gelfoam®-embedded hMSCs at both time points (Figure 3(a)). There was a significant upregulation of 1.8- and 1.2-fold difference for OPN and RUNX2 expression, respectively, when Gelfoam®-embedded hMSCs progressed from day 7 to 21. Even though the relative change is slightly less than that observed in the control cultures, the changes in OPN and RUNX2 verified that hMSCs embedded in Gelfoam® do undergo osteogenesis with time and that the presence of Gelfoam® did not affect their osteogenic potential.

### 3.4. Specific Integrin Proteins May Mediate Cell Adherence and Osteodifferentiation of hMSCs on Gelfoam®

We next evaluated the role of integrins (the major genes encoding for cell adhesion proteins), if any, in cell attachment and subsequent osteogenic differentiation processes. Using the parameters validated for the control cells (hMSCs undergoing osteogenic differentiation on tissue culture substrate), the expression profile of integrin subunits *α*2, *α*3, *α*5, *α*6, *β*1, *β*3, and *β*5 at days 7 and 21 after osteogenic induction of hMSCs embedded in Gelfoam® were analyzed (Figure 3(b)). As cells differentiated from day 7 to day 21, all the integrin subunits except *α*2 and *α*5 maintained relatively consistent expression profiles. The consistent expression throughout the differentiation process is evident with the fold level changes close to 1. The *α*2 subunit showed a significant upregulation over the course of differentiation, suggesting it to be the major cell adhesion protein; while *α*5 was downregulated indicating that it may not be involved in osteogenesis. Results suggest that the adherence, and potentially the osteogenic differentiation, of hMSCs on Gelfoam® could be mediated via specific integrin subunits.

### 3.5. Rat as an Animal Model to Evaluate hMSCs in a Tooth Extraction Defect

After confirming cytocompatibility of Gelfoam® and verifying that the material does not impede the osteogenic capacity of seeded hMSCs, we next implanted cell-seeded constructs in a rat maxillary alveolar tooth extraction defect model. All rats recovered quickly after surgery and returned to drinking, eating, and grooming within 48 hours. During the experimental period, the rats exhibited normal behavior without any weight loss or postoperative complications.

### 3.6. Histomorphometric Analyses

**Figure 4:**
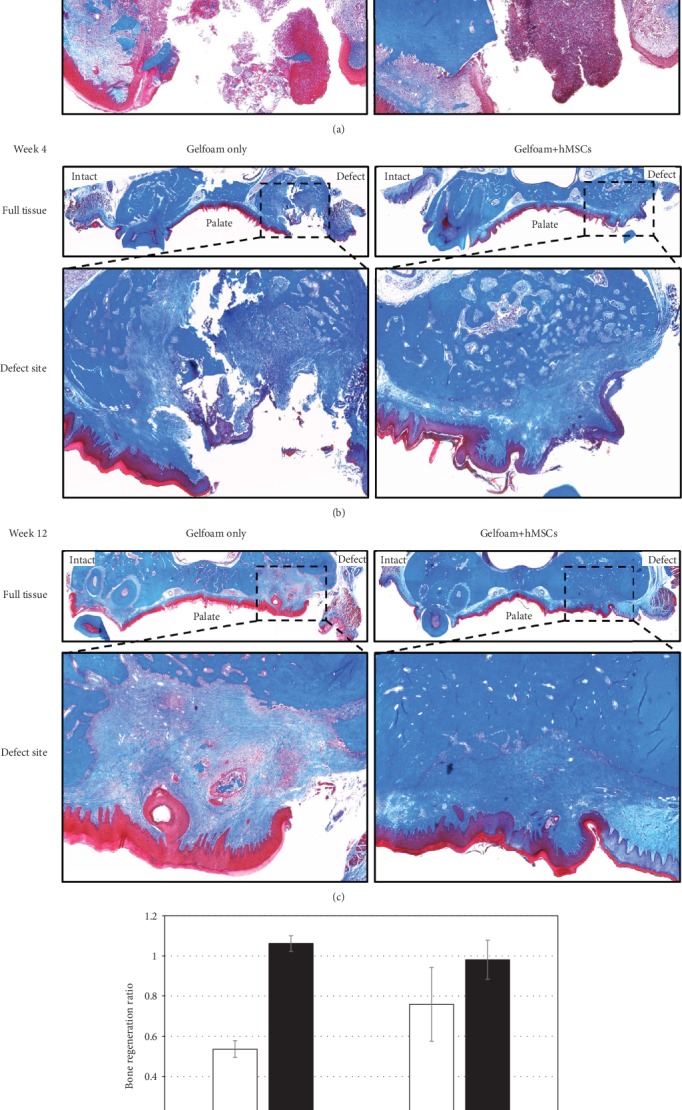
Qualitative and qualitative histomorphometry. Representative images depicting Masson trichrome stained coronal plane of the alveolar bone regions from rats treated with Gelfoam® alone and Gelfoam®+hMSCs for 1 (a), 4 (b), and 12 (c) weeks are shown. The intact and the defect sites are labelled in full tissue images, and the defect region has been expanded below respective images. Bone regeneration ratio values (d) from images demonstrate the level of bone formation within the defect site between week 1 and weeks 4 and 12, respectively, for both Gelfoam® alone and Gelfoam®+hMSCs. There is a statistically significant bone regeneration in 4 weeks in the presence of hMSCs.

Special stains of H&E and Masson trichrome were used for histomorphometric analyses. As anticipated and verified by the examination of H&E-stained slides, there was no evidence of adverse reaction due to either Gelfoam® or the hMSCs when implanted *in vivo.* Masson trichrome staining was evaluated for the formation of early new bone and filling of the defects. Representative images from samples containing Gelfoam® alone and those containing Gelfoam®+hMSCs are shown (Figure 4(a)). The top panels of these images show the entire maxillary region, with the intact tooth on the left and the defect on the right side, to aid in understanding the orientation and the anatomy of the rat maxilla, whereas the lower panels show a high-resolution image of the defect subjective assessment of the Masson trichrome staining of treatment groups which showed no significant bone formation by week 1, yet soft tissue in each defect, shown in red, was apparent. Early collagen and bone formation structures attempting to refill the defect were observed at 4 weeks in the rats treated with Gelfoam® alone. Though osteoblast activity was apparent in these samples, the majority of new tissue did not appear to be solid/mineralized and instead presented as a loose and irregular connective tissue. Rats treated with Gelfoam®+hMSCs in contrast, demonstrated defects that appeared to be filled largely with structures indicating solid/mineralized bone by week 4. As some regions of the perceived bone formation lacked mineralization, this suggests that osteoblast activity was still underway. At week 12 in rats treated with Gelfoam® only, defects had been completely filled, yet the light blue to purple color of the stained tissue indicated incomplete loose bone tissue formation. Comparatively, Gelfoam®+hMSC-treated defects demonstrated complete filling of the defect region with mineralized bone at week 12. Taken together, it is evident that the rats that received Gelfoam®+hMSCs showed significantly higher filling of the defect and new bone formation starting at week 4 and progressing into week 12.

The Masson trichrome-stained samples illustrated in Figure 4(a) were quantitated and analyzed and the data is shown in Figure 4(b). No significant differences were observed at any time point in the rats treated with Gelfoam® alone. In contrast, there were significant differences in early bone formation/collagen in rats treated with Gelfoam®+hMSCs between weeks 1 and 4 and between weeks 1 and 12. There was no statistical difference in bone regeneration between weeks 4 and 12. The hMSC-treated rats showed quantitatively more consistent accumulation of collagen/early bone formation structures than the rats treated with Gelfoam® alone. It appeared that the regeneration process started as early as 4 weeks. Furthermore, and most importantly, the level of bone formation between weeks 1 and 4 was roughly 2-fold significantly higher in the rats treated with Gelfoam®+hMSCs compared to the group with Gelfoam® alone. Comparatively, the level of bone formation between weeks 1 and 12 was not statistically different between the two treatment groups, suggesting an enhanced and early bone healing in presence of hMSCs.

### 3.7. Immunohistochemical Assessment

**Figure 5:**
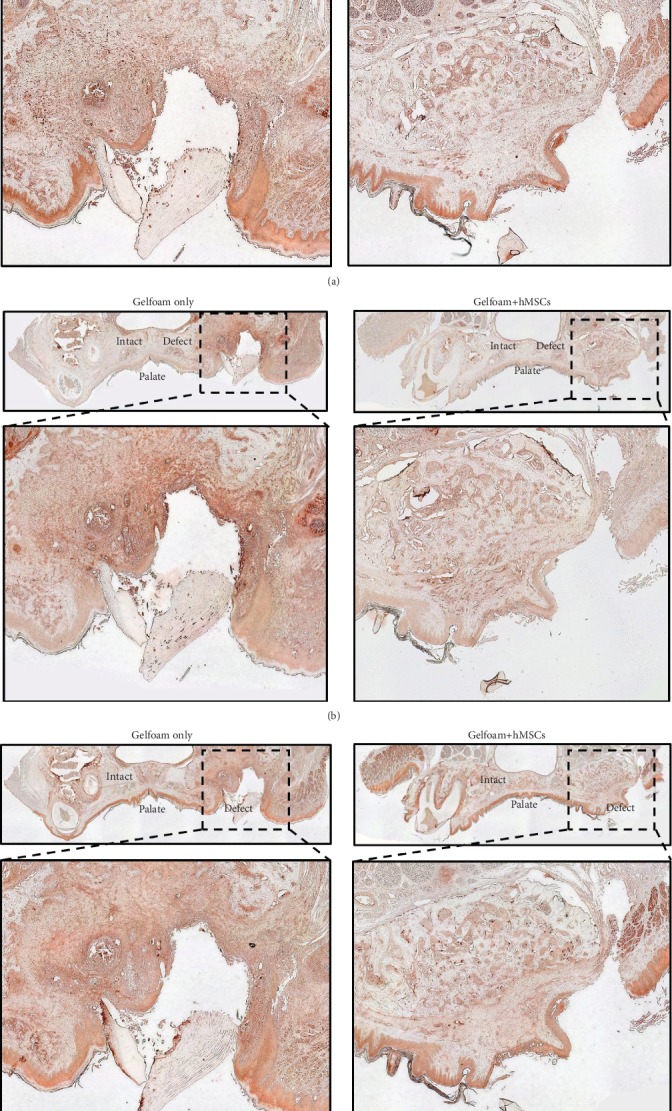
Immunohistochemistry. Representative images depicting immunohistochemical staining (Nove Red) of decalcified bone samples with OPN (a), FN (b), and CD34 (c) are shown. Histological sections from rats treated with Gelfoam® alone and Gelfoam®+hMSCs for 4 weeks are shown. The anatomical regions are labelled. Black dotted lines indicate region of interest illustrated in the 10x magnification. Note the areas of relatively organized pattern of staining in the hMSC-treated defects.

IHC evaluation of unstained histological sections for osteopontin (OPN) (Figure 5(a)), fibronectin (FN) (Figure 5(b)), and CD34 (Figure 5(c)) verified expression of these proteins within the tissue, for all rats at week 4, supporting the healing of the bone defect observed and described in the Masson trichrome-stained samples. Notable expression of OPN was observed in the soft tissue covering the palatal side as well as within the center of defects. Morphological comparison of surfaces stained for OPN within defects appeared to demonstrate a more uniform patterning within the Gelfoam®+hMSC-treated samples, in contrast to the chaotic formation in the Gelfoam®-only treated defects. FN expression was observed to be concentrated in the soft tissue covering defects, similar to OPN staining, as well as throughout the defects, indicating matrix formation within treated regions. Similar to morphological observations in OPN-stained samples, FN appeared more uniformly organized in the Gelfoam®+hMSC-treated sample as compared to the Gelfoam®-only treatment group. CD34 expression was heavily pronounced in both the treatment groups, within the defects and the surrounding tissue indicating presence of hematopoietic cells.

## 4. Discussion

Relative to bone marrow, the adipose tissue is a commonly used source of MSCs for oral/maxillofacial surgeons in bone tissue engineering [24]. The stromal vascular fraction of the adipose tissue is one of the commonly used sources of MSCs, and hence, an important tissue to regenerative medicine scientists and researchers [7, 25]. Adipose tissue-derived MSCs can be isolated relatively easily with less pain to the donor and in greater quantities. MSCs express specific markers (CD29, CD44, CD90, and CD105), demonstrate adipogenic, osteogenic, and chondrogenic potentials and enhancement of angiogenesis and immunomodulatory function, and have been used in the repair and regeneration of craniomaxillofacial injuries. Multiple reports have been published demonstrating the use of MSCs in the repair of calvarial and mandibular bone defects in rodent models [26–28]. Relatively speaking, there are less reports on tooth extraction models in rats and mouse models. This can partially be attributed towards the technical challenges associated with creating the defect and postop care in a small rodent model.

In the *in vitro* and *in vivo* assays described in this study, the bioinert scaffold, Gelfoam®, was used to deliver and contain hMSCs to the injury site. Gelfoam® is generally available and commonly used in the medical field as a hemostat. Known to be a compressible, porous, pliable, water insoluble, sponge-like material with absorptive properties, Gelfoam® also is completely absorbed by soft tissues in four to six weeks with little or no tissue reaction. We demonstrated that the material is cytocompatible with hMSCs and that cells are not hindered in the adherence, proliferation, or osteogenic potential when seeded on constructs. Though scaffolds became spongy after hydration by the media (*in vitro*) and body fluids (*in vivo*), the matrix appeared to maintain its structure throughout the study period. As Gelfoam® is not considered to be bioactive, the observed osteogenic differentiation of seeded cells is attributed to exposure to a 3D environment, which readily offers a mode of cell adhesion and permits multidirectional growth as compared to that observed along the 2D surface of the tissue culture polystyrene dish. This 3D growth pattern is more favorable to the formation of nodular cell clusters, which are hallmarks of cell osteodifferentiation [29].

Though alizarin red staining is considered to be the gold standard to evaluate *in vitro* osteogenesis, it could not be used in this study due to nonspecific absorbance of the stain by Gelfoam®, and as a result, evaluation was carried out utilizing the expression of osteogenic-specific genes to confirm cell adhesion and osteogenic differentiation. OPN is known to be expressed in osteoblasts during bone formation and remodeling [30]. RUNX2 is a transcription factor that is expressed during osteoblast differentiation and potentially upregulates the expression of bone matrix proteins [31]. The expression profiles of OPN and RUNX2 indicated the osteogenic capacity of the hMSCs seeded on Gelfoam®.

Similarly, the expression profiles of the various integrin subunits during osteogenesis were interesting. Integrin expression is known to be a relevant biomarker of successful cell adhesion. Integrins are also important in signal transduction during differentiation and osteogenesis [32, 33]. Integrins exist and are functional in the cells as heterodimers of alpha and beta subunits, and hence, it was necessary to evaluate the expression of these subunits independently. The minor changes in expression levels from day 7 to day 21 of the integrin subunits in the hMSCs embedded in Gelfoam® indicate that all the subunits, primarily *α*2 and not *α*5 are needed in the adhesion and subsequent osteogenic differentiation of hMSCs. Overall, the gene expression profile of the integrin subunits and the osteogenic genes of hMSCs embedded in Gelfoam® indicated that cell adhesion and osteogenic capabilities were not affected by Gelfoam®, and hMSCs exhibit normal molecular and cellular properties, further confirming that the material was cytocompatible.

A relatively complex and challenging rat model was used to evaluate the *in vivo* osteogenic potential of hMSCs delivered within a bioinert vehicle. This model offered a means of initial *in vivo* assessment of biocompatibility and regeneration potential of MSCs as rats tolerate the xenogenic implantation of MSCs very well. Previous studies from our laboratory demonstrate lack of immunologic or an adverse reaction when MSCs of xenogenic (goat or equine) origin are implanted in Sprague Dawley rats [34, 35]. Even though this model is convenient, cost effective, and considered to be ideal to test the performance of new implant and grafting materials in pretranslational studies, challenges due to the small animal size and the complex anatomy of the oral/maxillofacial region were apparent. Despite these challenges, histomorphometric data analyses showed that there was a statistically significant increase in the level of bone formation within 4 weeks when the defects were treated with hMSCs. This significance was observed relative to the defects treated with the Gelfoam scaffold alone, and proved our hypothesis. Subjective evaluation of the Masson trichrome staining revealed a more consistent and organized pattern of solid bone tissue regeneration in the group treated with hMSCs. This was further supported by IHC assessment of samples from both study groups at week 4 postsurgery. Stainings illustrated the presence of key osteogenic, cell matrix, and angiogenic proteins within the defect region at this time point. The Gelfoam®+hMSC-treated samples demonstrated a more organized morphological distribution of these proteins compared to the chaotic pattern in Gelfoam® alone samples. Our data strongly suggests that xenogenic adipose tissue-derived MSCs exhibit a potential to regenerate bone when delivered and contained using a scaffold. Future studies with large animal models are necessary to validate observations and elucidate mechanism(s) responsible for induced healing and repair of bone defects by MSCs.

## 5. Conclusions

We have demonstrated that xenogeneic hMSCs, delivered and contained at the bone injury site via a bioinert scaffold, promoted enhanced regeneration of maxillary bone defects. The relative availability and ease of collection for adipose-derived MSCs coupled with the observed osteogenic potential when applied and maintained within a bone defect presents a promising bioactive additive for bone tissue engineering materials. Application of such cell-based material platforms therefore offers a feasible and effective approach for the clinical restoration of oral/maxillofacial bone defects.

## Data Availability

The data used to support the findings of this study are available from the corresponding author upon request.

## Abbreviations

FN: Fibronectin
H&E: Hematoxylin and eosin
hMSCs: Human mesenchymal stem cells
HRP: Horseradish peroxidase
IACUC: Institutional Animal Care and Use Committee
ONJ: Osteonecrosis of the jaw
MSCs: Mesenchymal stem cells
OPN: Osteopontin
ROI: Region of interest.

## Additional Points

*Availability of Data and Material*. The datasets used and/or analyzed during the current study are available from the corresponding author on reasonable request.
